# Supplementary figures and images for: Genetic background influences survival of infections with Salmonella enterica serovar Typhimurium in the Collaborative Cross
Source: PLoS Genet. 2022 Apr 13;18(4):e1010075. doi: 10.1371/journal.pgen.1010075 (PMC9067680; doi:10.1371/journal.pgen.1010075)

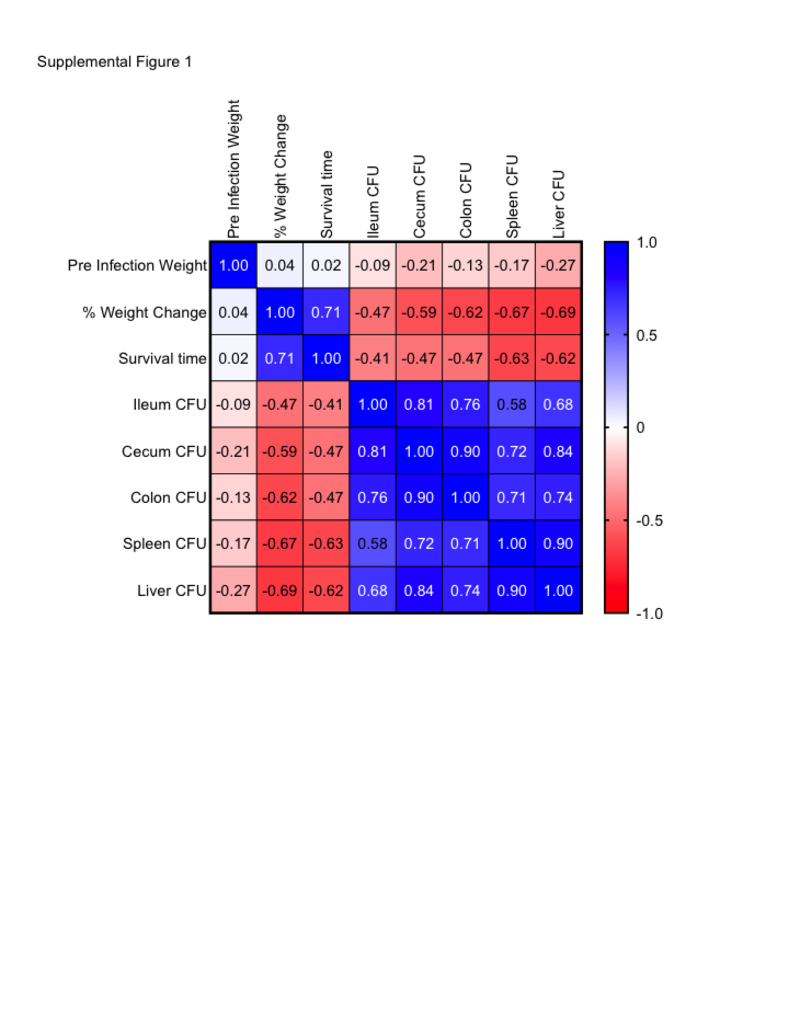

Supplement: S1 Fig — Strain medians for 32 CC strains were analyzed using Spearman correlation. Blue denotes positive correlations and red denotes negative correlations. (TIF) [file pgen.1010075.s001.tif]

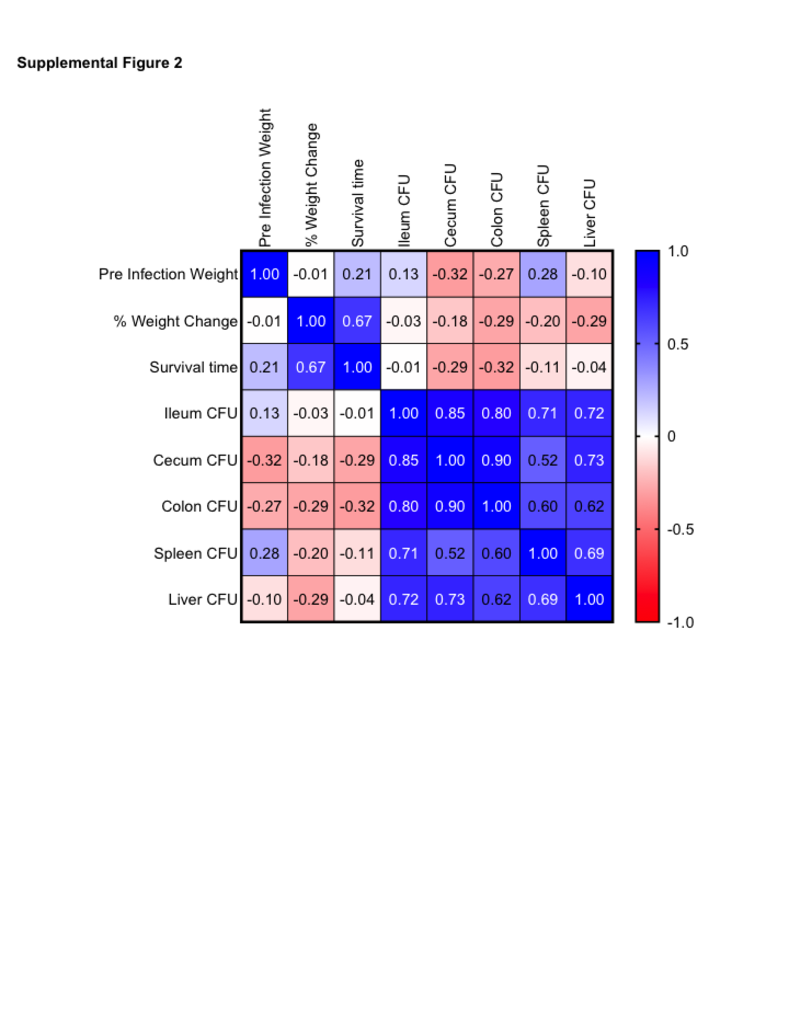

Supplement: S2 Fig — Strain medians for 14 CC strains that are susceptible were analyzed using Spearman correlation. Blue denotes positive correlations and red denotes negative correlations. (TIF) [file pgen.1010075.s002.tif]

Supplemental Figure 3

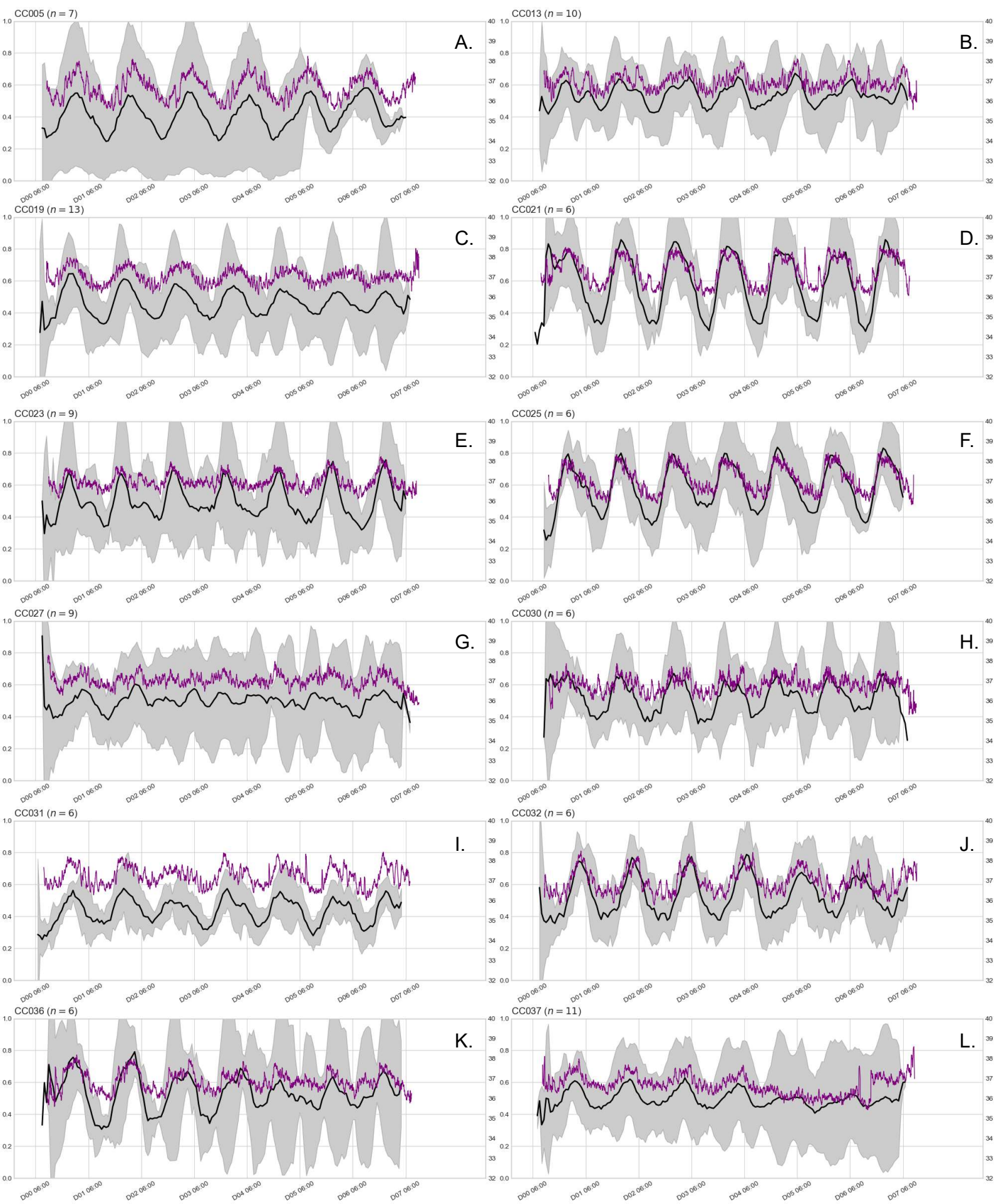

## Supplemental Figure 3 cont.

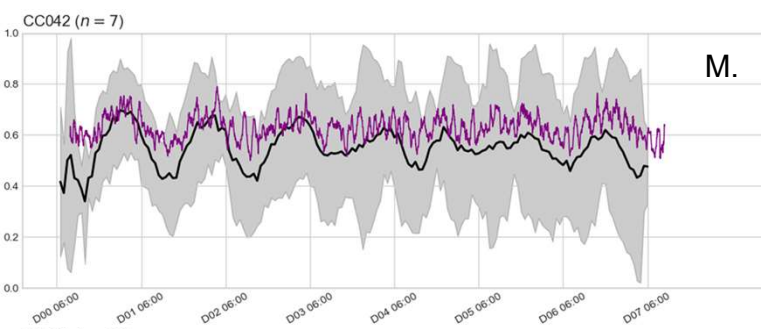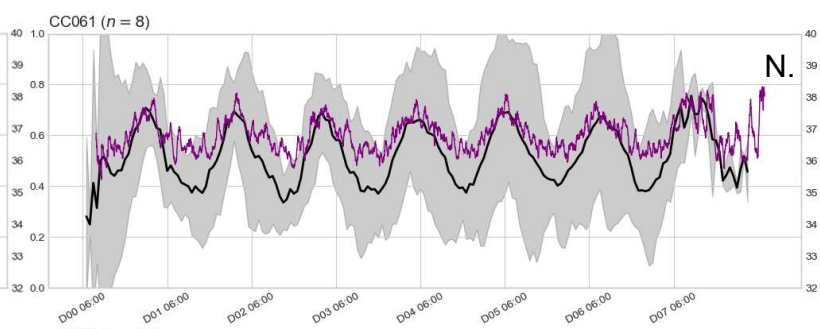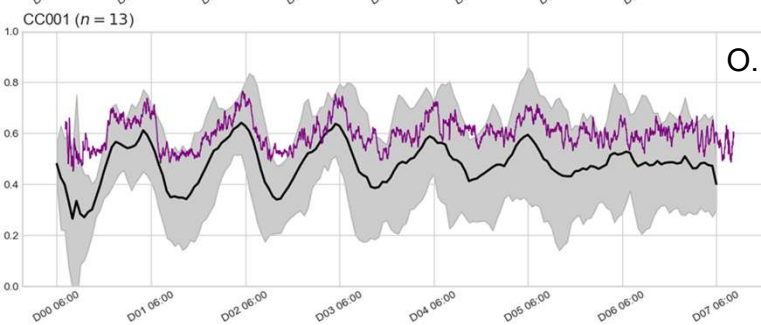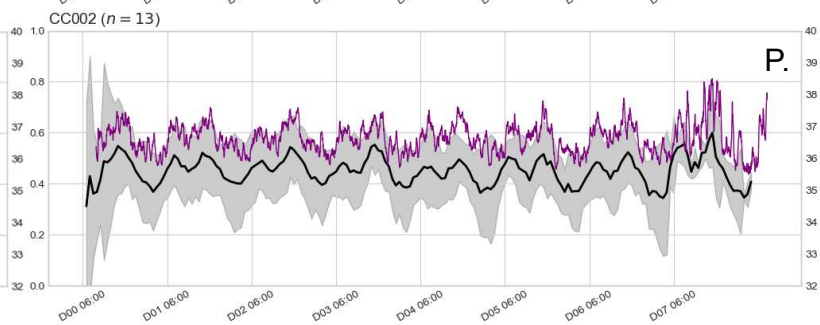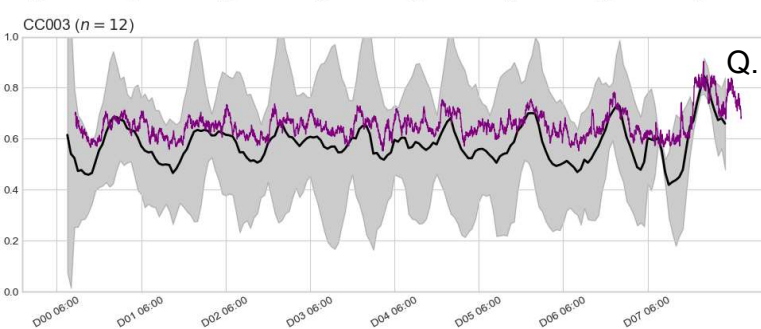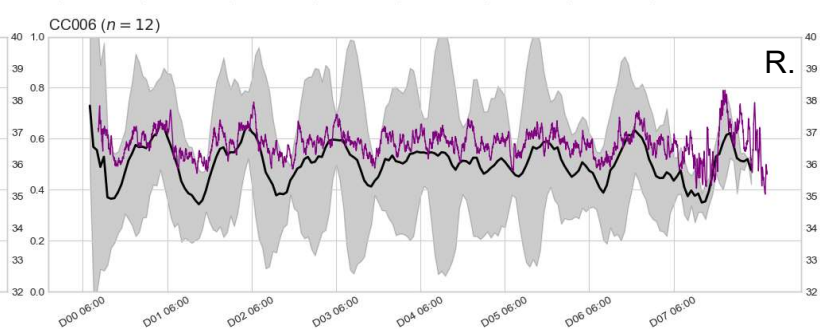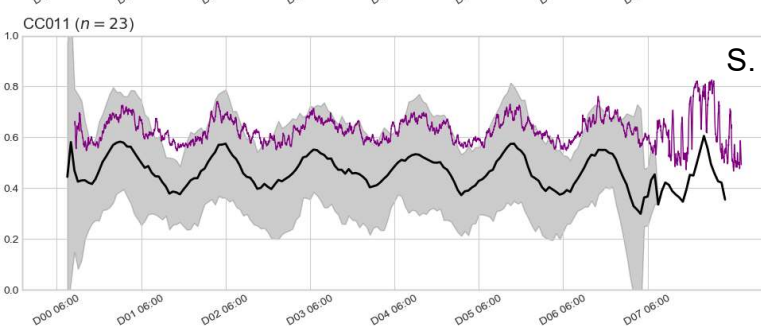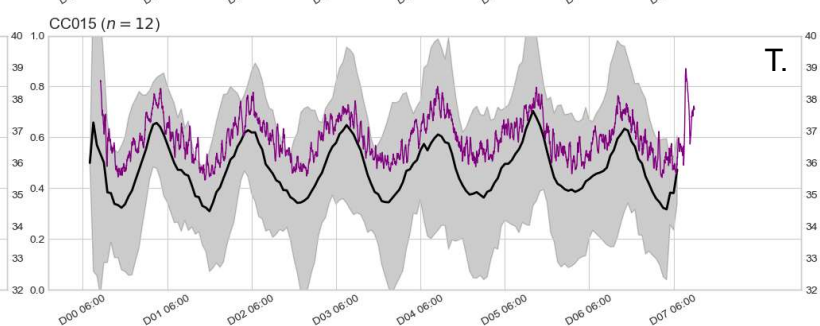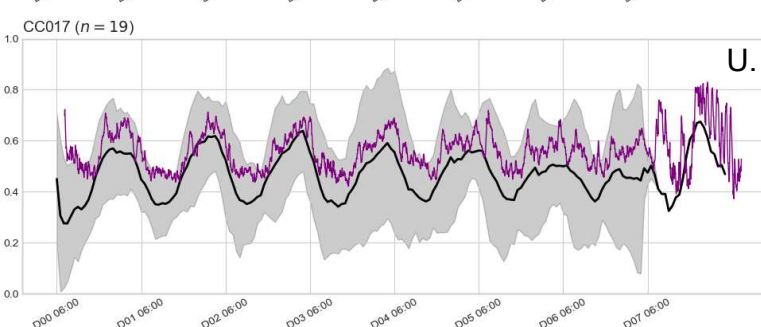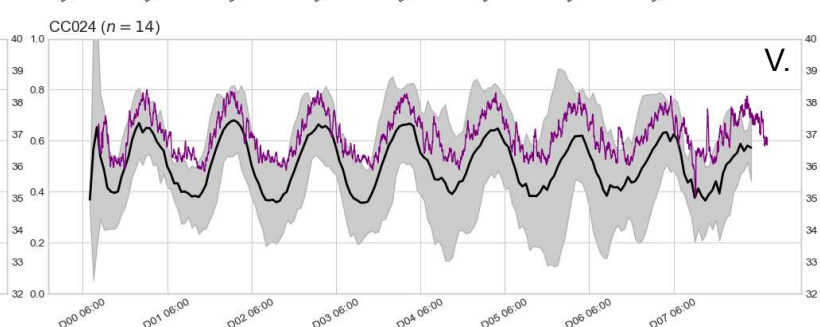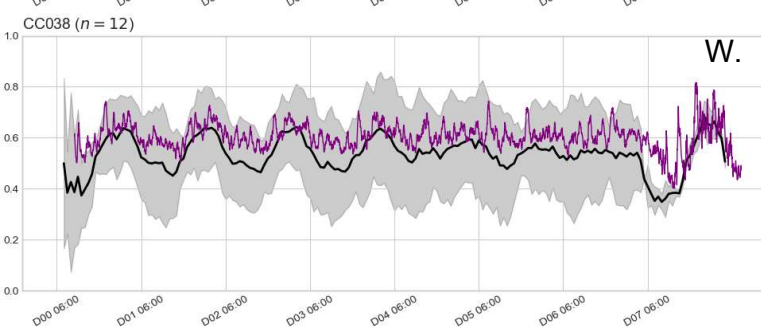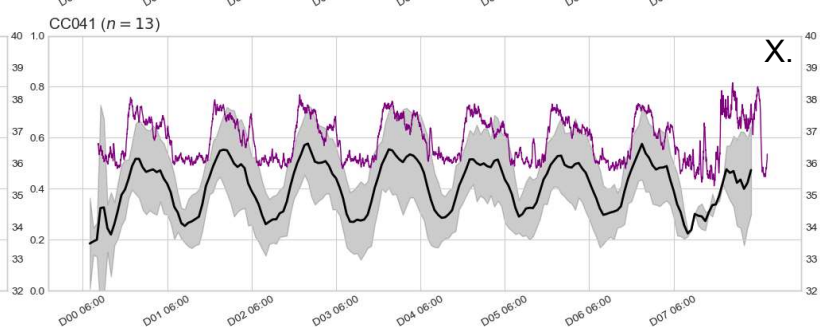

Supplemental Figure 3 cont.

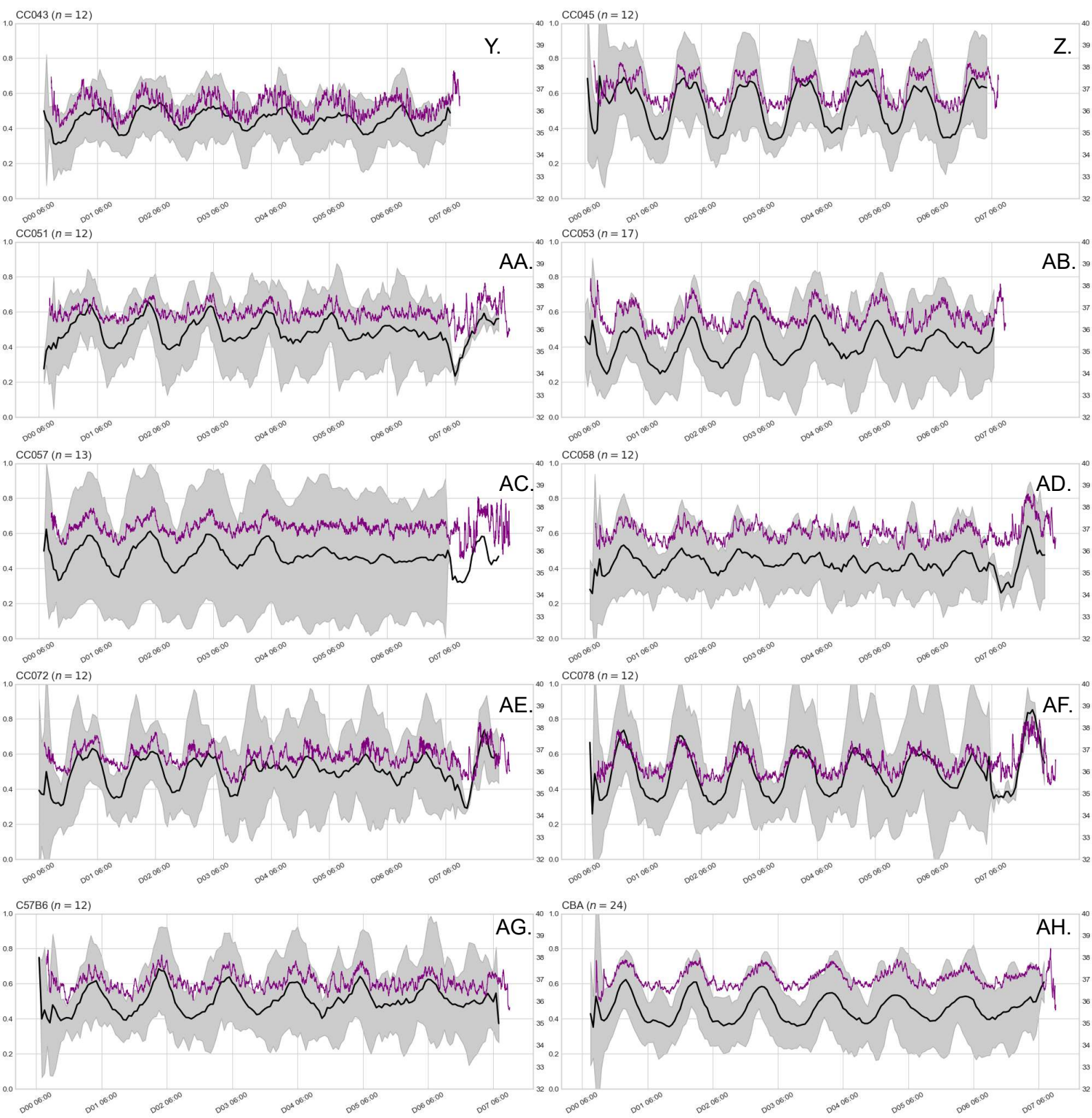

Supplement: S3 Fig — Purple lines represent the average temperature and black lines represent the average fraction of time the mouse is active pre-infection for 7 days. The gray area represents +/- 2 standard deviations. A-N are susceptible strains, O-AF are surviving strains, and AG-AH are control strains. (A) Panels A-L, (B) Panels M-X, and (C) Panels &Y-AH. (PDF) [file pgen.1010075.s003.pdf]

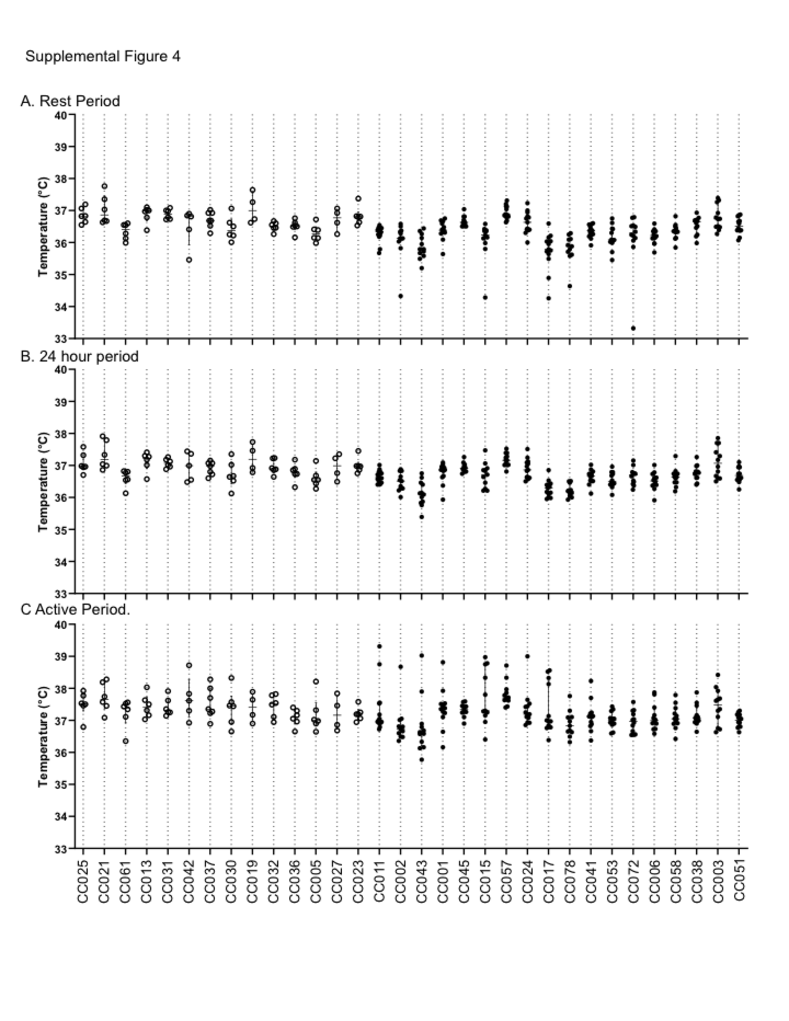

Supplement: S4 Fig — A. Minimum, B. median, and C. maximum baseline body temperatures for 32 CC strains corresponding to the rest period, 24-hour period, and active period. Susceptible strains represented by open circles and surviving strains by closed circles. Median and Interquartile range shown by lines. (TIF) [file pgen.1010075.s004.tif]

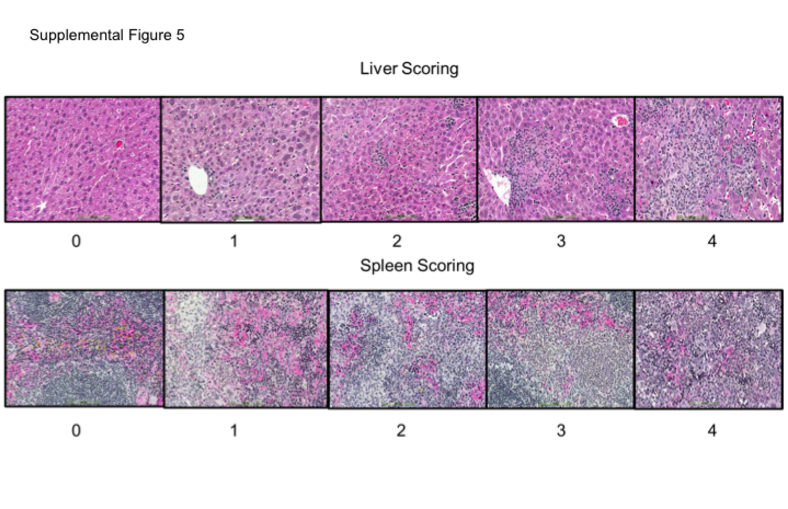

Supplement: S5 Fig — Tissues were sectioned and stained with H&E before being analyzed. Images from Dr. L. Garry Adams illustrating scoring matrix in S1 Table. (TIF) [file pgen.1010075.s005.tif]

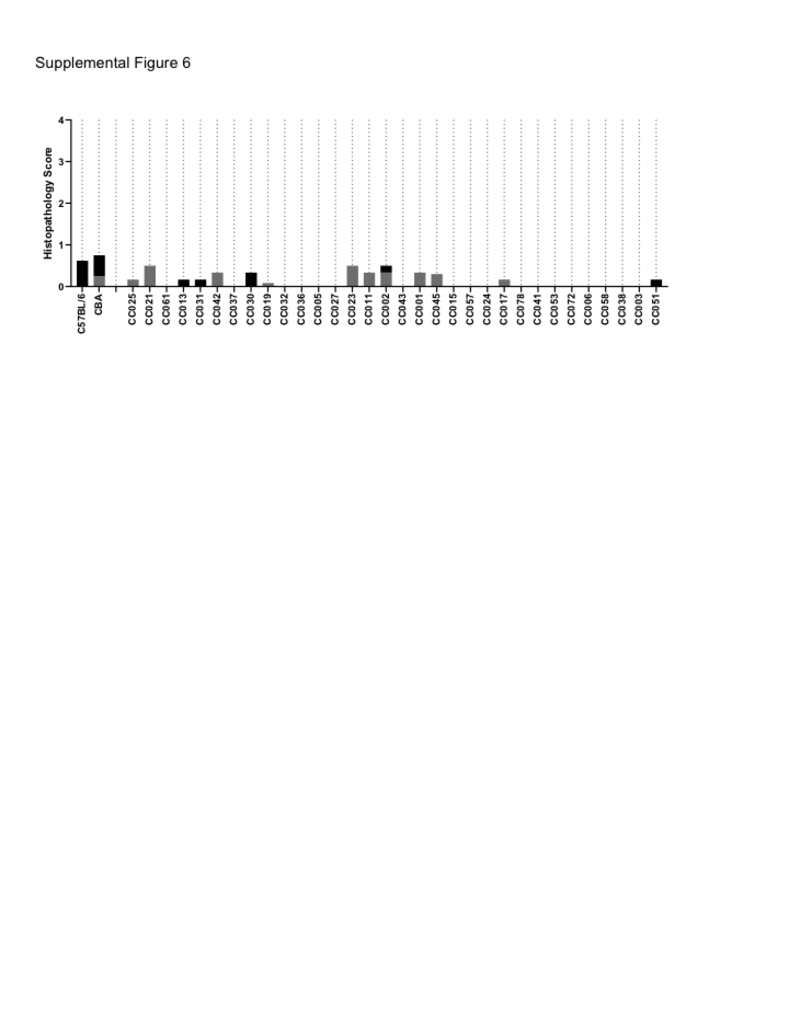

Supplement: S6 Fig — Median ileum (gray) and cecum/colon (black) histopathology scores. Scored on a logarithmic scale of 0 to 4, with 0 being normal tissue and 4 being severely damaged tissue. (TIF) [file pgen.1010075.s006.tif]

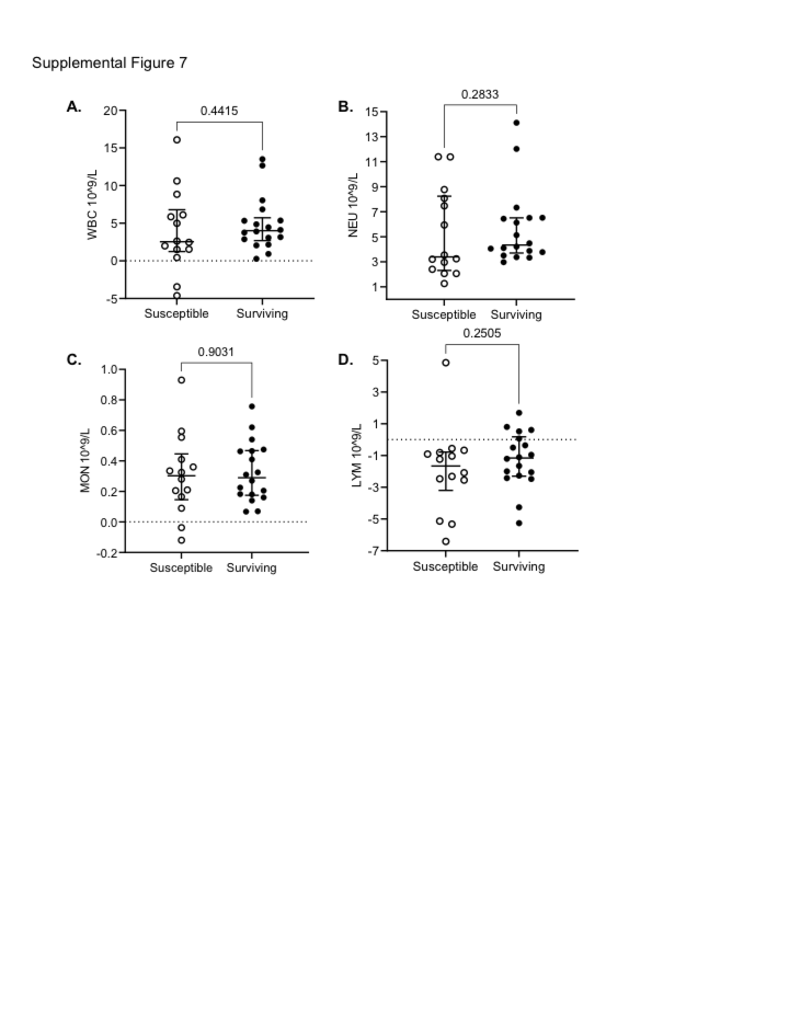

Supplement: S7 Fig — Strain medians for change in blood count (infected-uninfected) for A. total white blood cells, B. neutrophils, C. monocytes, and D. lymphocytes, grouped by survival status. A two-tailed Mann-Whitney test was used to determine statistical significance. (TIF) [file pgen.1010075.s007.tif]

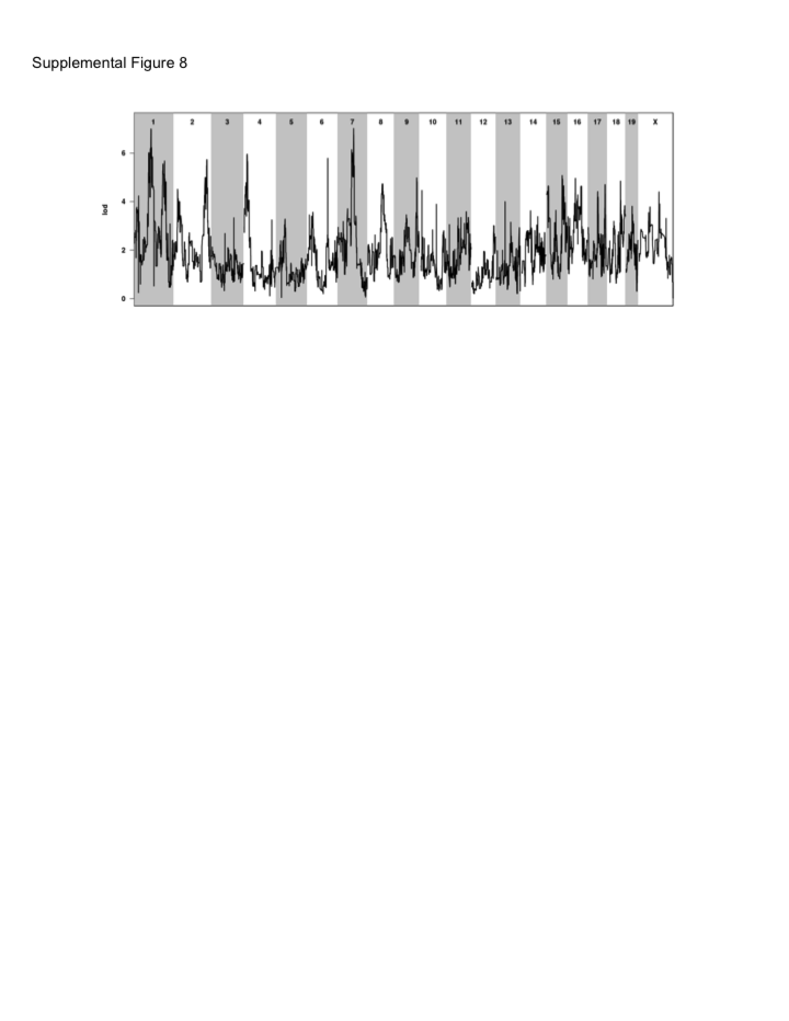

Supplement: S8 Fig — This value defines how rapidly each strain succumbed to infection. QTL analysis included 30 CC strains, excluding CC042 and CC045. (TIF) [file pgen.1010075.s008.tif]

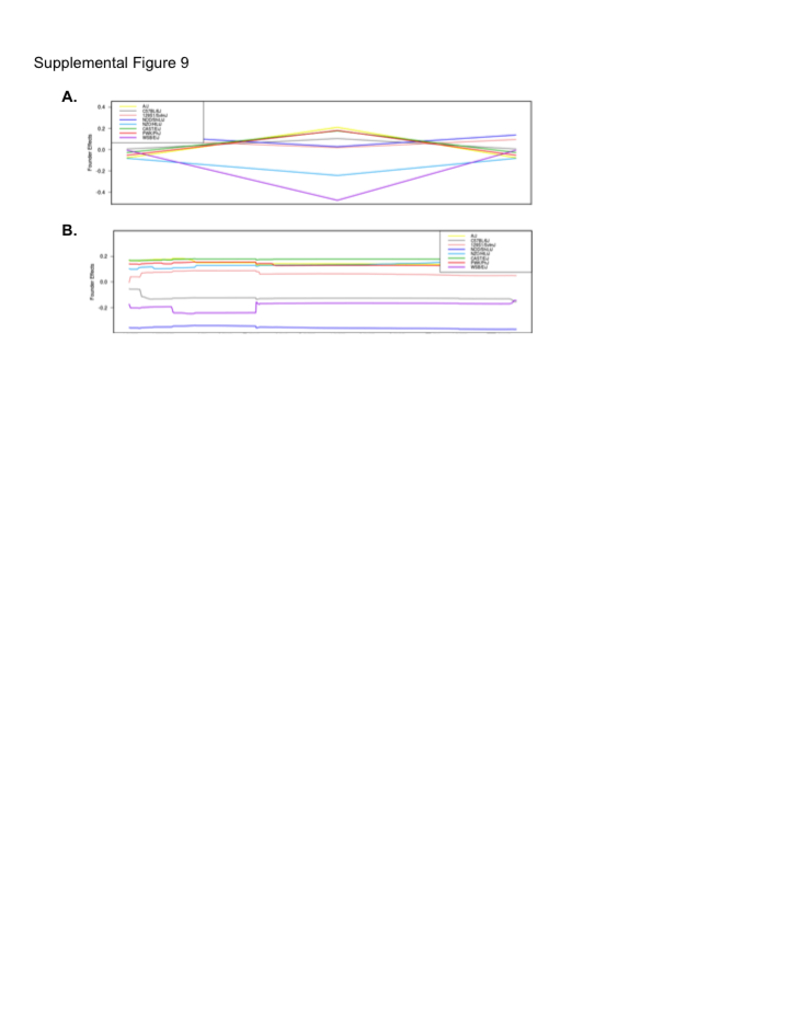

Supplement: S9 Fig — 30 CC strains included, after removal of CC042 and CC045. Founder effect plots for A. Chr 6 and B. 7 focused on the QTL peak, genes within the region are also shown. Results obtained using gQTL analysis. (TIF) [file pgen.1010075.s009.tif]

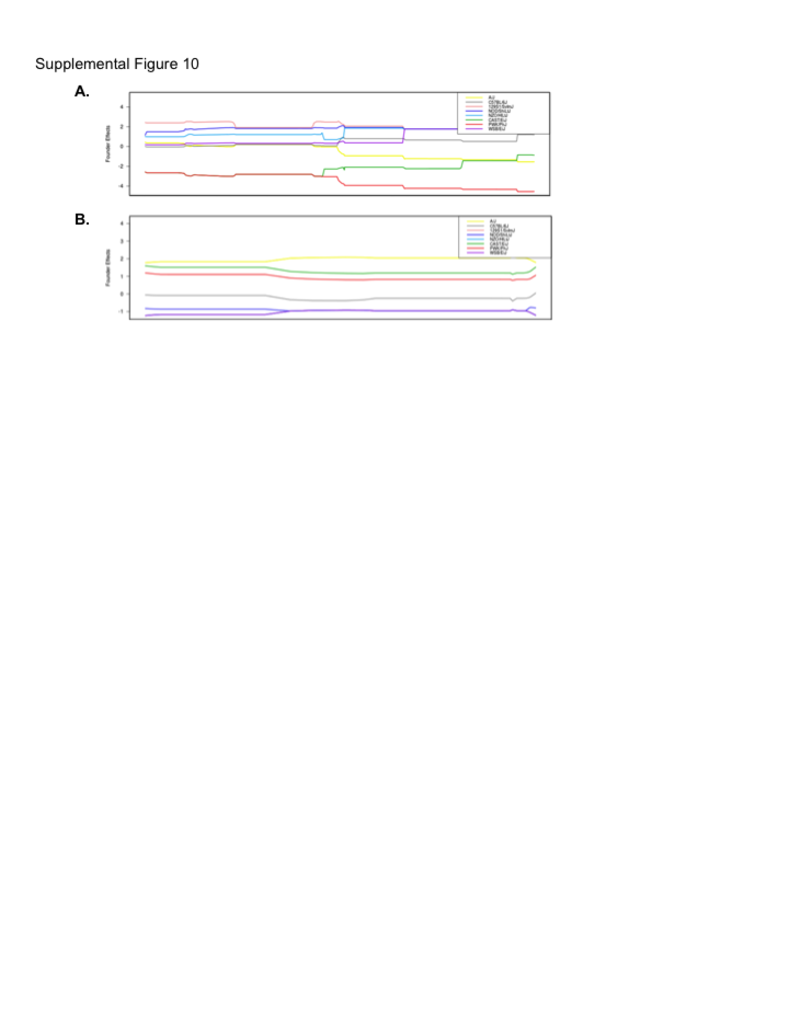

Supplement: S10 Fig — Analysis included 30 CC strains, after removal of CC042 and CC045. Founder effect plots for A. Chr 2 and B. 4 focused on the QTL peak, genes within the region are also shown. Results obtained using gQTL analysis. (TIF) [file pgen.1010075.s010.tif]

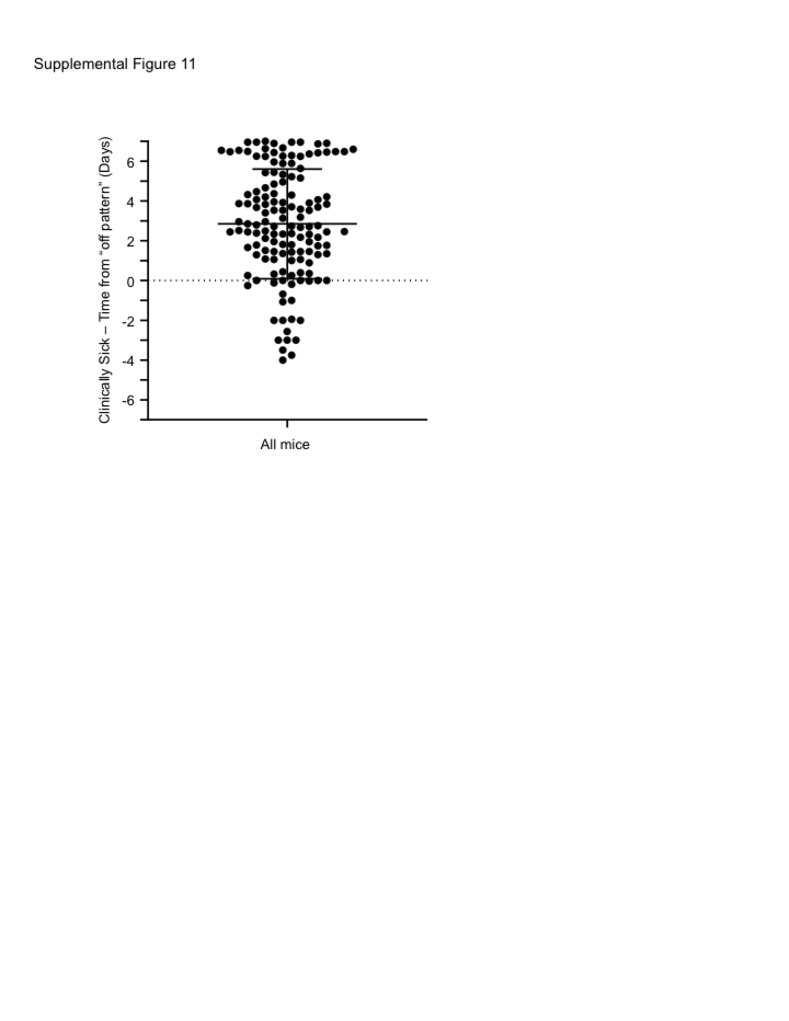

Supplement: S11 Fig — Time from first change in temperature pattern subtracted from time that mouse developed visually observable clinical signs of disease. Mean and standard deviation shown by strains and individual mice shown by dots. (TIF) [file pgen.1010075.s011.tif]

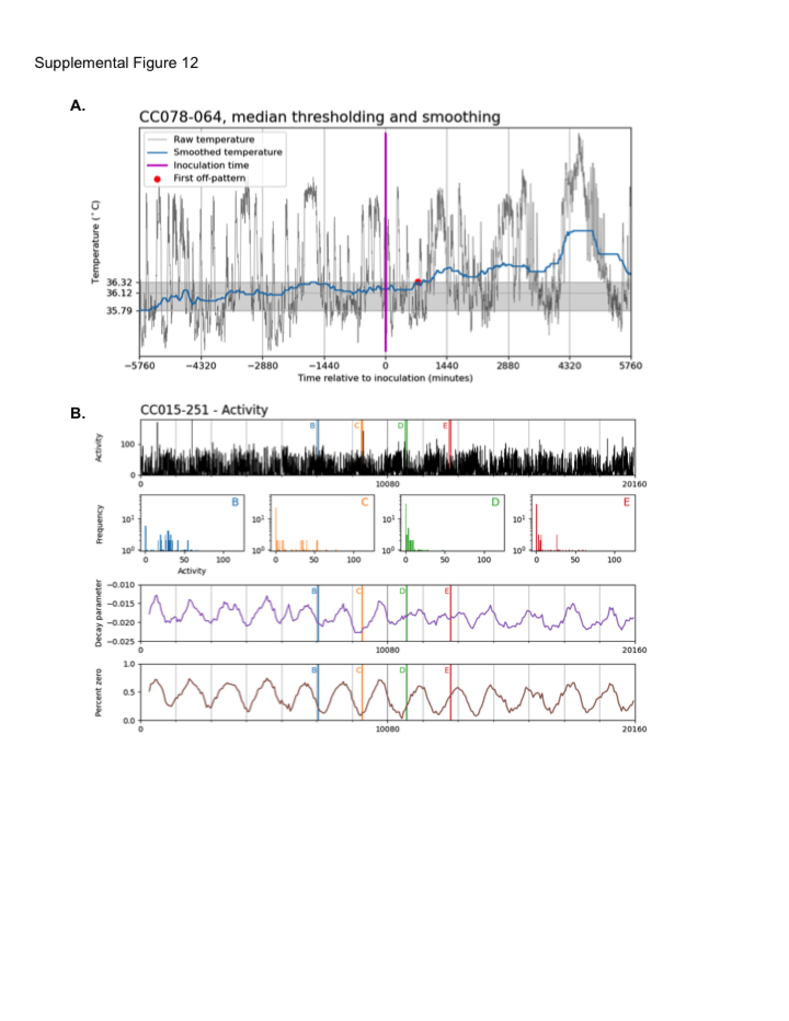

Supplement: S12 Fig — Examples of how A. temperature and B. activity calculations were determined. Specifically maximum, median, minimum pre-infection temperature and activity as well “off-pattern” for post-infection. (TIF) [file pgen.1010075.s012.tif]

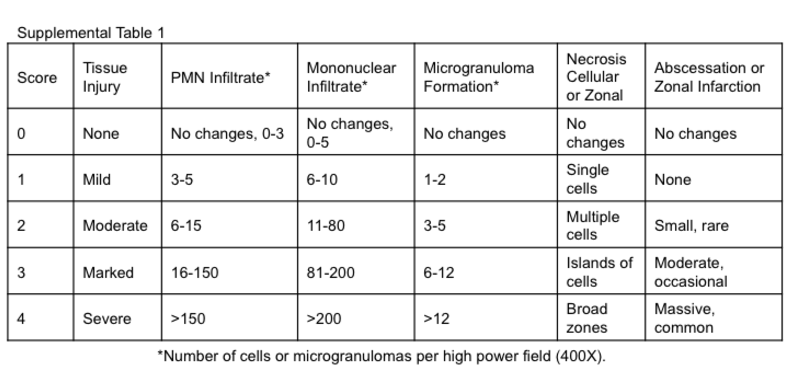

Supplement: S1 Table — Final score per organ corresponds to the highest score assigned to a single category. Scoring matrix from Dr. L. Garry Adams. (TIFF) [file pgen.1010075.s013.tiff]

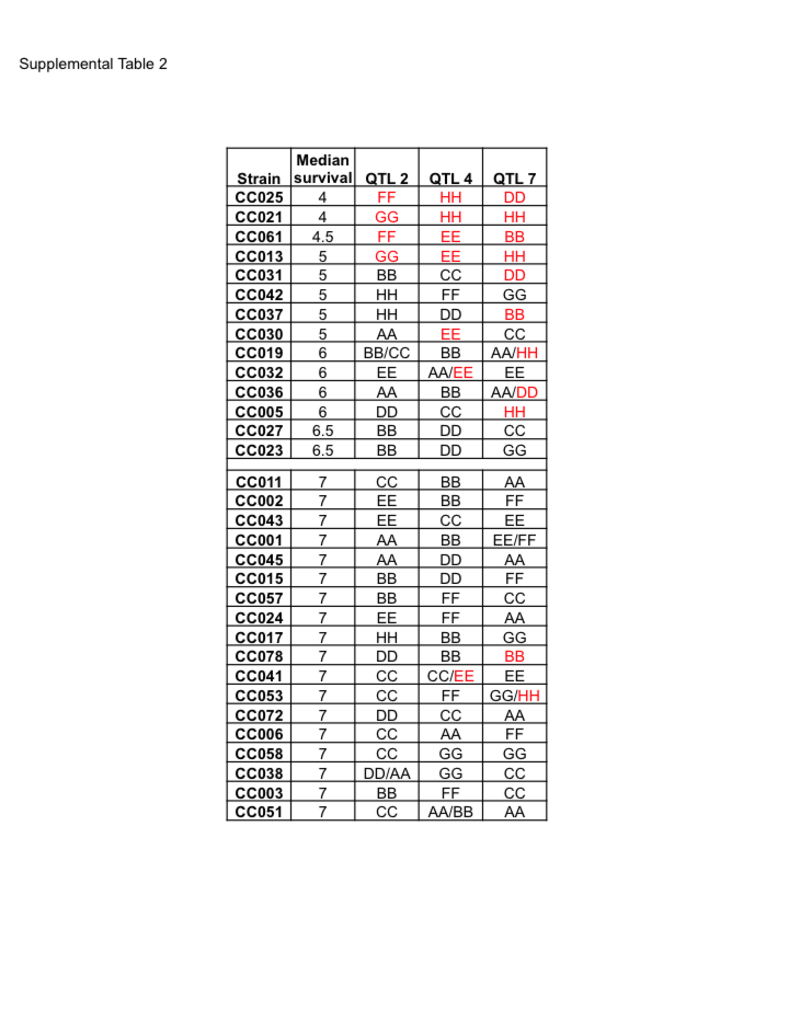

Supplement: S2 Table — All 32 CC strain haplotypes for the three QTL regions shown. Red letters have a low haplotype effect and are linked to poor survival. AA—A/J, BB–C57BL/6J, CC– 129SvlmJ, DD–NOD/ShiLtJ, EE—NZO/HlLtJ, FF—CAST/EiJ, GG—PWK/PhJ, HH—WSB/EiJ. (TIF) [file pgen.1010075.s014.tif]

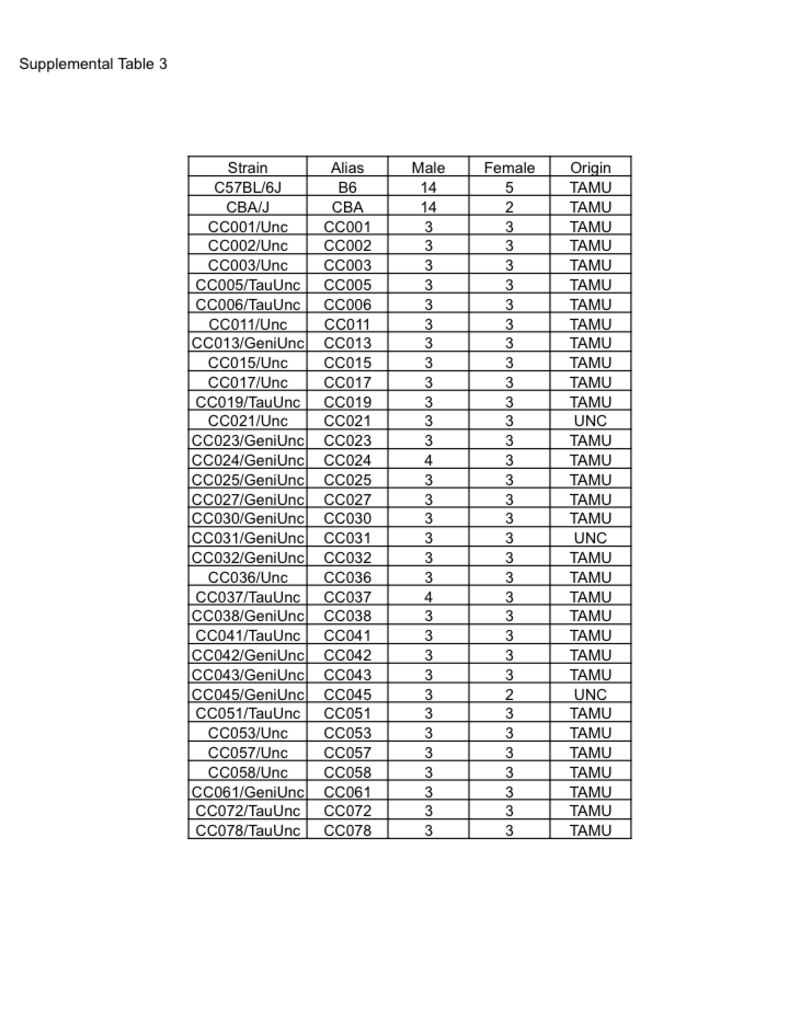

Supplement: S3 Table — (TIF) [file pgen.1010075.s015.tif]
